# Supplementary figures and images for: Probiotic Bifidobacterium longum supplied with methimazole improved the thyroid function of Graves’ disease patients through the gut-thyroid axis
Source: Commun Biol. 2021 Sep 7;4:1046. doi: 10.1038/s42003-021-02587-z (PMC8423791; doi:10.1038/s42003-021-02587-z)

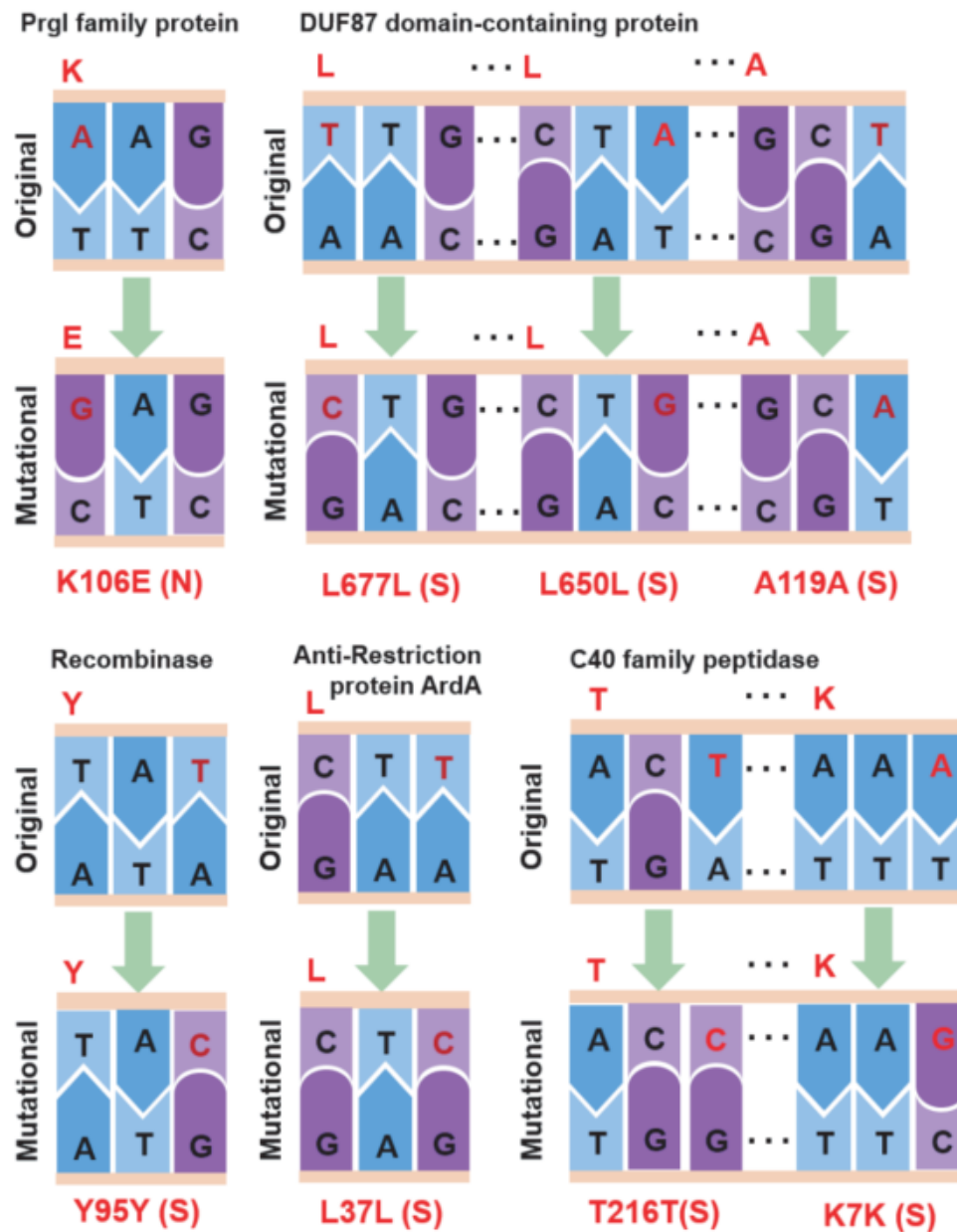

Figure S1. SNP annotated in *Roseburia hominis* genome

Supplement: Supplementary file 2 — Supplementary Information [file 42003_2021_2587_MOESM2_ESM.pdf]
