# Supplementary material for: Probiotic Bifidobacterium longum supplied with methimazole improved the thyroid function of Graves’ disease patients through the gut-thyroid axis
Source: Commun Biol. 2021 Sep 7;4:1046. doi: 10.1038/s42003-021-02587-z (PMC8423791; doi:10.1038/s42003-021-02587-z)
Supplement: Supplementary file 3 — Description of Additional Supplementary Files [file 42003_2021_2587_MOESM3_ESM.pdf]

Description of Additional Supplementary Files

File name: Supplementary Data 1

Description: Subjects' basic information

File name: Supplementary Data 2

Description: Subjects' clinical indexes
