# Supplementary material for: Probiotic Bifidobacterium longum supplied with methimazole improved the thyroid function of Graves’ disease patients through the gut-thyroid axis
Source: Commun Biol. 2021 Sep 7;4:1046. doi: 10.1038/s42003-021-02587-z (PMC8423791; doi:10.1038/s42003-021-02587-z)
Supplement: Supplementary file 6 — Reporting Summary [file 42003_2021_2587_MOESM6_ESM.pdf]

nature research

Corresponding author(s): Jiachao Zhang
Last updated by author(s): 2021-04-16

Reporting Summary

Nature Research wishes to improve the reproducibility of the work that we publish. This form provides structure for consistency and transparency in reporting. For further information on Nature Research policies, see our Editorial Policies and the Editorial Policy Checklist.

Statistics

For all statistical analyses, confirm that the following items are present in the figure legend, table legend, main text, or Methods section.

n/a	Confirmed	
☐	☒	The exact sample size (n) for each experimental group/condition, given as a discrete number and unit of measurement
☐	☒	A statement on whether measurements were taken from distinct samples or whether the same sample was measured repeatedly
☐	☒	The statistical test(s) used AND whether they are one- or two-sided<br>*Only common tests should be described solely by name; describe more complex techniques in the Methods section.*
☐	☒	A description of all covariates tested
☐	☒	A description of any assumptions or corrections, such as tests of normality and adjustment for multiple comparisons
☐	☒	A full description of the statistical parameters including central tendency (e.g. means) or other basic estimates (e.g. regression coefficient) AND variation (e.g. standard deviation) or associated estimates of uncertainty (e.g. confidence intervals)
☒	☐	For null hypothesis testing, the test statistic (e.g. F, t, r) with confidence intervals, effect sizes, degrees of freedom and P value noted<br>*Give P values as exact values whenever suitable.*
☒	☐	For Bayesian analysis, information on the choice of priors and Markov chain Monte Carlo settings
☒	☐	For hierarchical and complex designs, identification of the appropriate level for tests and full reporting of outcomes
☐	☒	Estimates of effect sizes (e.g. Cohen's d, Pearson's r), indicating how they were calculated

Our web collection on statistics for biologists contains articles on many of the points above.

Software and code

Policy information about availability of computer code

Data collection	All of the 75 DNA samples were subjected to shotgun metagenomic sequencing by using an Illumina HiSeq 2500 instrument in the Novogene Company (Beijing, China). Libraries were prepared with a fragment length of approximately 300 bp. Paired-end reads were generated using 100 bp in the forward and reverse directions. The quality of the reads were controlled by FastQC and were subsequently aligned to the human genome to remove the host DNA fragments.
Data analysis	All statistical analyses were performed using the R software. PCoA analysis was performed in R using the "ade4" package. CLR transformation was performed by the "zCompositions" package. Heatmaps were constructed using the "pheatmap" package. The differential abundances of various profiles were tested with the Wilcoxon rank-sum test and were considered significantly different at p < 0.05. For boxplot construction, the package "ggpubr" was used. The edges of the network were calculated based on the determined Spearman's rank correlation coefficients and visualized in Cytoscape v3.7.141. The potential mechanism figure was constructed by using an online tool named "BioRender".<br>The sequence data reported in this paper have been deposited in the NCBI database (resequencing and metagenomic sequencing data: PRJNA693409).<br>All the project analysis code had been deposited in GitHub: https://github.com/zhjch321123/GD_Probiotic_treatment

For manuscripts utilizing custom algorithms or software that are central to the research but not yet described in published literature, software must be made available to editors and reviewers. We strongly encourage code deposition in a community repository (e.g. GitHub). See the Nature Research guidelines for submitting code & software for further information.

Data

Policy information about availability of data

All manuscripts must include a data availability statement. This statement should provide the following information, where applicable:
- Accession codes, unique identifiers, or web links for publicly available datasets
- A list of figures that have associated raw data
- A description of any restrictions on data availability

The sequence data reported in this paper have been deposited in the NCBI database (resequencing and metagenomic sequencing data: PRJNA693409).
All the project analysis code had been deposited in GitHub: https://github.com/zhjch321123/GD_Probiotic_treatment

Field-specific reporting

Please select the one below that is the best fit for your research. If you are not sure, read the appropriate sections before making your selection.

☒ Life sciences ☐ Behavioural & social sciences ☐ Ecological, evolutionary & environmental sciences

For a reference copy of the document with all sections, see nature.com/documents/nr-reporting-summary-flat.pdf

Life sciences study design

All studies must disclose on these points even when the disclosure is negative.

Sample size	A total of 75 fecal samples were included in the present research.
Data exclusions	Not applicable
Replication	Not applicable
Randomization	Not applicable
Blinding	Not applicable

Reporting for specific materials, systems and methods

We require information from authors about some types of materials, experimental systems and methods used in many studies. Here, indicate whether each material, system or method listed is relevant to your study. If you are not sure if a list item applies to your research, read the appropriate section before selecting a response.

Materials & experimental systems

n/a	Involved in the study	
☒	☐	Antibodies
☒	☐	Eukaryotic cell lines
☒	☐	Palaeontology and archaeology
☒	☐	Animals and other organisms
☐	☒	Human research participants
☒	☐	Clinical data
☒	☐	Dual use research of concern

Methods

n/a	Involved in the study	
☒	☐	ChIP-seq
☒	☐	Flow cytometry
☒	☐	MRI-based neuroimaging

Human research participants

Policy information about studies involving human research participants

Population characteristics	In the present study, all GD patients were recruited from the Hainan Provincial People's Hospital, Haikou, China.<br>The subjects' basic information (gender, age, BMI, smoking, and alcoholism) and clinical indexes were recorded in Table S1.
Recruitment	They were divided into 3 groups including the methimazole treatment group (GA, n=8), the methimazole+black-bean treatment group (GB, n=9), and the methimazole+probiotic (Bifidobacterium longum) treatment group (GC, n=8), all subjects stayed on their treatment for 6 months.

Ethics oversight	The study was reviewed and approved by the Ethics Committee of the Hainan Provincial People's Hospital (2018-109), and informed consent was obtained from all volunteers in written form before they were enrolled in the study. Sampling and all described subsequent steps were conducted in accordance with the approved guidelines.

Note that full information on the approval of the study protocol must also be provided in the manuscript.
